# Neuropsychiatric disorders among Ecuadorian patients with multiple sclerosis and neuromyelitis optica spectrum disorder

**DOI:** 10.3389/fneur.2025.1587804

**Published:** 2025-07-11

**Authors:** Edgar Patricio Correa-Díaz, Ruth Jimbo Sotomayor, Jorge Rubén Pilco Romero, Patricio Alexander Merino Aguilera

**Affiliations:** ^1^Department of Neurology, Hospital de Especialidades Carlos Andrade Marín, Quito, Ecuador; ^2^Pontifical Catholic University of Ecuador, Quito, Ecuador; ^3^Center for Research for Health in Latin America (CISEAL), Quito, Ecuador; ^4^Central University of Ecuador, Quito, Ecuador; ^5^Department of Internal Medicine, Hospital Oncológico Solón Espinosa Ayala, Quito, Ecuador

**Keywords:** multiple sclerosis, optical neuromyelitis, neuropsychiatric disorders, anxiety, depression, psychosis, bipolar disorder

## Abstract

**Introduction:**

Multiple Sclerosis (MS) and Neuromyelitis Optica Spectrum Disorder (NMOSD) are chronic demyelinating diseases of the central nervous system, there is an association between these diseases and neuropsychiatric disorders, which directly impacts the course of the disease and the quality of life of the patients.

**Objective:**

To determine the prevalence of psychiatric disorders in patients with MS and NMOSD and its relationship with age and disability. Materials and methods: It is a cross-sectional study, 151 patients were included, 117 patients with MS and 34 patients with NMOSD. Information was obtained by collecting data from records of the patients. In order to establish the diagnoses of anxiety and depression, the Hamilton Depression Rating Scale (HDRS) and the Beck Depression Inventory (BDI) scales were completed.

**Results:**

MS and NMOSD were frequent in female sex and mestizo ethnicity. The psychiatric disorders were frequent in both diseases (70.9% in MS and 85% in NMOSD), the most frequent pathologies were anxiety and depression. We found low frequency of attempted suicide. Anxiety and depression were present in patients between 41 and 50 years of age. There is not a correlation between the severity of anxiety and depression with age and disability.

**Conclusion:**

It is the first Ecuadorian study in which we established a high prevalence of anxiety and depression in MS and NMOSD patients, the severity of both disease was not related with age and disability.

## 1 Introduction

Multiple Sclerosis (MS) and Neuromyelitis Optica Spectrum Disorder (NMOSD) are immune-mediated chronic inflammatory demyelinating diseases affecting the central nervous system (1, 2). There is an association between neuropsychiatric disorders and demyelinating diseases, which directly impacts the course of the disease as well as the quality of life. Neuropsychiatric pathologies usually appear during the course of the demyelinating diseases, but up to 1% of patients start with them (3). Previous studies have showed that neuropsychiatric features were present in 80 to 95% of MS patients (4). In NMOSD the presence of neuropsychiatric comorbidities is also frequent a systematic review has demonstrated that the prevalence of depression and anxiety is 40–45%, respectively (5). However, evidence from LATAM is scarce. One study from Argentina has demonstrated that the prevalence of psychiatric comorbidities is higher in NMOSD than in MS patients (6, 7).

Anxiety and depression are frequent in MS patients, a meta-analysis showed that the prevalence of these disorders were 22 and 30%, respectively. According to the DSM-5, depression is characterized by “persistent low mood, lack of positive affect, and loss of interest in normally pleasant activities, usually for more than 2 weeks,” while that generalized anxiety disorder is an “excessive worry and anxiety that are difficult to control, causes anguish and significant deterioration, most of the days for at least 6 months.” These diseases generate inflammatory and degenerative changes that cause a variety of symptoms which compromise the self-care and independence of patients, it can affect social and work relationships (4, 8, 9). In NMOSD the prevalence of depression and anxiety is also frequent previous studies have showed a prevalence of 40 and 28%, respectively (10, 11). Bipolar Disorder is less common in MS and NMOSD, however, the prevalence is twice that of the general population (12).

Regarding the prevalence of drug abuse in patients with demyelinating diseases, it is low between 2.5 to 7.4%. One example is the cannabis consumption in MS patients in order to control spasticity, pain, tremor and bladder dysfunction. Alcohol abuse is also infrequent in MS patients (3.9 to 18.2%) (4). In LATAM few studies have evaluated the presence of neuropsychiatric disorders in demyelinating diseases and in Ecuador there are no reports on psychiatric comorbidities in MS and NMOSD (6, 13). For this reason, the objective of this study is to establish the prevalence of neuropsychiatrist disorders in an Ecuadorian cohort of patients with demyelinating disorders and its relationship with age and disability.

## 2 Methods

### 2.1 Clinical and demographic assessment

A cross-sectional and descriptive study was carried out at Carlos Andrade Marín Hospital in the city of Quito, Ecuador, which is a referral center for patients with MS and NMOSD. Currently, 60% of patients with MS and 70% of patients with NMOSD diagnosed in Ecuador are attended at this center. We enrolled MS and NMOSD patients who met the 2017 McDonald criteria and 2015 international consensus criteria, respectively. We did not include patients with myelin oligodendrocyte glycoprotein antibody-associated disease (MOGAD) as we do not have resources for testing antibodies against myelin oligodendrocyte glycoprotein (MOG).

Demographic data including gender, age and years of education was collected. All patients were offered detailed clinical assessment including rating of disability on the Expanded Disability Status Scale (EDSS). According EDSS, we define mild disability when EDSS was <3,5, moderate disability with EDSS between 4 to 6 and severe disability when EDSS was >6,5 (14).

### 2.2 Mood disorder evaluation

In order to establish the diagnoses of anxiety and depression, the Hamilton Depression Rating Scale (HDRS) and the Beck Depression Inventory (BDI) scales were completed. The Hamilton Anxiety Scale (15), evaluates 14 points: anxious mood, tension, fears, insomnia, intellect, depressed mood, muscular discomfort, sensory discomfort, cardiovascular, respiratory, gastrointestinal, genitourinary, autonomic symptoms, and interview behavior with a total score ranging from 0 to 56. A score of 0 to 5 indicates absence of anxiety, 6 to 14 mild anxiety, and equal to or higher than 15 moderate/severe anxiety.

The Beck Depression Inventory (BDI-II) consists of 21 questions with a total score ranging from 0 to 40. A score of 0 to 9 points means that the patient does not present depression, 10 to 18 points is mild depression, 19 to 29 points moderate depression, and equal to or higher than 30 points is severe depression (16). The Hamilton Anxiety Scale and BDI-II scales have been previously validated in Ecuador (17, 18).

Regarding to the evaluation of bipolar disorder, the Beck Depression Scale (BDI-II) was applied, which was previously discussed; in addition, the Mini International Neuropsychiatric Interview (MINI) scale was used, an instrument used to confirm neuropsychiatric diagnoses and to evaluate the patient’s current symptoms (19). It is carried out through a structured interview of short duration, approximately 15 min, which evaluates the main psychiatric disorders; this has been updated with a new module to assess the specifier with mixed features for manic and hypomanic episodes of the DSM-V, divided into 16 modules, specifically, the modules for major depressive episode, psychotic disorders and manic/hypomanic disorders were used. The MINI scale was previously validated in the city of Quito-Ecuador with the Gamboa-Proaño study (20).

Additionally, the Bipolar Disorder Course Assessment Scale (CGI-BP-M Scale) was used, which is composed of three subscales, two of which assess the severity of acute symptoms of mania and depression; the third assesses the longitudinal severity of the disease, giving a score of 1 to 7, where 1 is normal and 7 is very severe (21), this scale validated in the Ecuadorian population previously (22).

The Suicidal Orientation Inventory (ISO-30 scale) was used to assess suicide risk (23), which was designed by King and Kowalchuk in 1994, it consists of 30 items that offer three categories, low suicidal risk (less than 30 points), moderate risk (31–44 points) and high suicidal risk (greater than 45 points). The ISO-30 Scale was also validated in Ecuador (24).

The psychiatric measures were administered by trained psychologists. Assessments were undertaken individually with each participant and lasted approximately 20 min.

All participants were more than 18 years and provided informed consent. The study was reviewed and approved by the Ethics Committee.

### 2.3 Statistical analysis

We created a data base which contained demographics, disability, clinical type, and treatment. Data were analyzed in a descriptive manner by measuring central tendency and dispersion [confidence interval (CI) and standard deviation (SD)]. We used proportions for the analysis of categorical variables. Comparisons between categorical variables were made by chi-square contingencies tables and the Fisher exact test of significance. Comparison of anxiety and depression scores between NMO and MS groups was undertaken using independent samples t-test. We used non-parametric test (Kruskal-Wallis) for comparisons between quantitative variables with non-normal distribution. All data analysis was carried out through the statistical program Microsoft Excel and the SPSS (Statistical Package for the Social Science) software.

## 3 Results

### 3.1 Clinical and demographic characteristics

One hundred fifty-one patients with demyelinating disorders have completed the assessments, 117 of them had MS and 34 NMOSD. The clinical and demographic characteristics are presented in Table 1. In MS, the female sex and the mestizo ethnic group were more frequent affected. The average age was 42.3 years. The average of EDSS was 2.5 (SD +/− 1.95), mild disability predominated in 64.96% (*n* = 76/117). In NMOSD, female sex and mestizo ethnic group were also more frequent affected. In contrast with MS, the average age was 49.8 years and the average of EDSS was 4.23 (SD +/− 1.73), moderate disability predominated in 61.76% (*n* = 21/34).

**Table 1:** Socio-demographic characteristics of MS and NMOSD patients.

Variable	MS (*n* = 117)	NMOSD (*n* = 34)	p*
	*n* (%)	*n* (%)	
Sex			
Male	34 (29.06%)	5 (14.71%)	0.09
Female	83 (70.94%)	29 (85.29%)	
Ethnic group			
Mestizo	103 (88.03%)	31 (91.18%)	0.53
Indigenous	0	0	-
African-American	0	1 (2.94%)	-
Caucasian	14 (11.97%)	0	0.03
Montubio	0	1 (2.94%)	-
Other	0	1 (2.94%)	-
Age			
Mean (SD+/−):	42,3,077 (12,71)	49,8,529 (13,24)	0.02
Median (RIQ):	42 (16–76)	48,5 (22–79)	0.34
Current age (years)			
<18	3 (2.56%)	0	0.08
18 to 30	21 (17.95%)	2 (5.88%)	0.21
31 to 40	29 (24.79%)	5 (14.71%)	0.18
41 to 50	31 (26.50%)	13 (38.24%)	0.39
51 to 60	23 (19.66%)	9 (26.47%)	0.29
>61	10 (8.55%)	5 (14.71%)	
Marital status			
Single	46 (39.32%)	5 (14.71%)	0.007
Married	47 (40.17%)	26 (76.47%)	0.001
Widowed	3 (2.56%)	1 (2.94%)	0.90
Divorced	16 (13.68%)	1 (2.94%)	0.08
Common-law marriage	5 (4.27%)	1 (2.94%)	0.72
Educational level			
Primary	2 (1.71%)	6 (17.65%)	0.002
High school	26 (22.22%)	10 (29.41%)	0.39
University	89 (76.07%)	18 (52.94%)	0.009
None	0	0	-
EDSS			
Mean (SD):	2,5,000 (1,95)	4,2,353 (1,73)	0.007
Median (IQR):	2 (0–7,5)	4 (0–7,5)	
Level of disability			
Mild (EDSS: 0–3,5)	76 (64.96%)	6 (17.65%)	0.004
Moderate (EDSS: 4–6)	34 (29.06%)	21 (61.76%)	0.004
Severe (EDSS: > 6,5)	7 (5.98%)	7 (20.59%)	0.01
Phenotype of MS			
RRMS	101 (86.32%)	-	-
SPMS	6 (5.13%)	-	-
PPMS	5 (4.27%)	-	-
CIS	4 (3.42%)	-	-
RIS	1 (0.85%)	-	-
AQP4 antibody in NMOSD			
Positive AQP4	-	23 (67.65%)	-
Negative AQP4	-	9 (26.47%)	-
Unknown AQP4	-	2 (5.88%)	-

NMOSD, Neuromyelitis Optica Spectrum Disorders; MS, Multiple Sclerosis; RRMS; Relapsing–Remitting Multiple Sclerosis; SPMS, Secondary-Progressive Multiple Sclerosis; PPMS, Primary-Progressive Multiple Sclerosis; CIS, Clinical isolated syndrome; RIS, Radiologically isolated syndrome; EDSS, Expanded Disability Status Scale; AQP4, Antibodies to aquaporin-4; SD, standard deviation; IQR, interquartile range. *=*p* < 0.05.

When we compared demographic characteristics between MS and NMOSD patients, we found that there were not white NMOSD patients (*p* = 0.03), the age average was higher in NMOSD than in MS (*p* = 0.02), the education level was higher in MS than NMOSD (0.007). The disability was lower in MS than NMOSD (*p* = 0.004), the severe disability was more frequent in NMOSD than MS (*p* = 0.001).

### 3.2 Neuropsychiatric pathologies associated with MS and NMOSD

Result of the structured psychiatrics interviews are presented in Table 2. In general, the prevalence of neuropsychiatric disorders in the both diseases were high, 70.9% (*n* = 83/117) in MS and 85.29% (*n* = 29/34) in NMOSD. Moderate and severe anxiety was more frequent in NMOSD patients than in MS patients (*p* = 0.003). The frequency of depression was similar between the both pathologies.

**Table 2:** Neuropsychiatric pathologies associated with MS and SNMOSD.

Psychiatric pathology	MS (*n* = 117)	NMOSD (*n* = 34)	p*
	No (%)	No (%)	
Hamilton Anxiety Scale			
No anxiety	42 (35.9%)	6 (17.65%)	0.045
Mild anxiety	39 (33.3%)	8 (23.53%)	0.28
Moderate/severe anxiety	36 (30.7%)	20 (58.82%)	0.003
Beck depression inventory			
No depression	52 (44.4%)	9 (26.4%)	0.06
Mild depression	37 (31.62%)	16 (47.06%)	0.09
Moderate depression	21 (17.95%)	5 (14.71%)	0.66
Severe depression	7 (5.98%)	4 (11.76%)	0.25
Alcoholism			
Occasional consumption	50 (42.74%)	-	-
Chronic alcoholism	1 (0.85%)	2 (5.9%)	0.70
Borderline personality disorder	1 (0.9%)	-	-
Adjustment disorder	6 (5.1%)	2 (5.9%)	0.86
Specific phobia	1 (0.9%)	1 (2.9%)	0.35
Insomnia	5 (4.3%)	3 (8.8%)	0.30
Bipolar disorder	2 (1.7%)	1 (2.9%)	0.65
Suicidal tendency	-	2 (5.9%)	-

NMOSD, Neuromyelitis Optica Spectrum Disorders; MS, Multiple Sclerosis; HACM-A, Hamilton Anxiety Scale; BDI, Beck depression inventory; BPD, Borderline personality disorder; AD, Adjustment disorder; BDD, Bipolar disorder. *=*p* < 0.05.

Regarding to MS patients, 33.3% (*n* = 39/117) of them presented mild anxiety, while 30.7% (*n* = 36/117) presented moderate/severe anxiety. Fifty-six percent of MS patients had depression, 31.62% (*n* = 37/117) showed mild depression, while 5.98% had severe depression (*n* = 7/117). Less than 1% suffered from chronic alcoholism. Borderline personality disorder, social phobia, insomnia and bipolar disorder were infrequent in this cohort.

In NMOSD patients, 58.8% (*n* = 20/34) had moderate/severe anxiety. Twenty-six percent did not have depression, 47% (*n* = 16/34) presented mild depression while 11.7% of NMOSD patients had severe depression (*n* = 4/34). Chronic alcoholism, social phobia, insomnia, and bipolar disorder were infrequent in this cohort.

### 3.3 Neuropsychiatric pathologies in relation to age groups

In MS, the largest number of cases with mild and moderate anxiety were between 41–50 years. Depression also predominated in the same age. In NMOSD, the majority of psychiatric comorbidities were found also between the ages of 41 to 50 years. However, no statistically significant differences were found in relation to age groups in any of the categories (Table 3).

**Table 3:** Neuropsychiatric disorders in relation to age groups.

Neuropsychiatric disorders (years)	<18	18 to 30	31 to 40	41 to 50	51 to 60	>61	*p* value
	*n* (%)	*n* (%)	*n* (%)	*n* (%)	*n* (%)	*n* (%)	
MS (*n* = 117)							
Anxiety severity							
Mild	-	8 (6.8%)	12 (10.2%)	9 (7.6%)	10 (8.5%)	-	0.11
Moderate/severe	1 (0.8%)	9 (7.6%)	5 (4.2%)	11 (9.4%)	6 (5.1%)	4 (3.4%)	0.43
Depression severity							
None	1 (0.8%)	7 (5.9%)	15 (12.8%)	16 (13.6%)	8 (6.8%)	5 (4.2%)	0.64
Mild	1 (0.8%)	5 (4.2%)	10 (8.5%)	9 (7.6%)	11 (9.4%)	1 (0.8%)	0.33
Moderate	-	8 (6.8%)	4 (3.4%)	4 (3.4%)	2 (1.7%)	3 (2.5%)	0.085
Severe	1 (0.8%)	1 (0.8%)	-	2 (1.7%)	2 (1.7%)	1 (0.8%)	0.26
Chronic alcoholism	-	-	1 (0.8%)	-	-	-	0.11
Borderline personality disorder	-	-	1 (0.8%)	-	-	-	-
Adjustment disorder	1 (0.8%)	3 (2.5%)	-	1 (0.8%)	1 (0.8%)	-	0.052
Specific phobia	-	-	-	1 (0.8%)	-	-	-
Insomnia	-	1 (0.8%)	-	1 (0.8%)	3 (2.5%)	-	0.28
Bipolar disorder	-	1 (0.8%)	-	1 (0.8%)	-	-	0.75
Suicidal tendency	-	-	-	-	-	-	-
NMOSD (*n* = 34)							
Anxiety severity							
Mild	-	1 (2.9%)	2 (5.8%)	3 (8.8%)	1 (2.9%)	1 (2.9%)	0.71
Moderate/severe	-	1 (2.9%)	1 (2.9%)	8 (23.5%)	6 (17.6%)	4 (11.7%)	0.39
Depression severity							
None	-	-	4 (11.7%)	3 (8.8%)	2 (5.8%)	-	0.03
Mild	-	1 (2.9%)	1 (2.9%)	6 (17.6%)	4 (11.7%)	4 (11.7%)	0.48
Moderate	-	-	-	3 (8.8%)	1 (2.9%)	1 (2.9%)	0.74
Severe	-	1 (2.9%)	-	1 (2.9%)	2 (5.8%)	-	0.27
Chronic alcoholism	-	-	1 (2.9%)	1 (2.9%)	-	-	0.61
Borderline personality disorder	-	-	-	-	-	-	-
Adjustment disorder	-	-	-	1 (2.9%)	-	1 (2.9%)	0.61
Specific phobia	-	-	1 (2.9%)	-	-	-	-
Insomnia	-	-	-	1 (2.9%)	1 (2.9%)	1 (2.9%)	0.84
Bipolar disorder	-	-	-	1 (2.9%)	-	-	-
Suicidal tendency	-	1 (2.9%)	-	1 (2.9%)	-	-	0.07

NMOSD, Neuromyelitis Optica Spectrum Disorders; MS, Multiple Sclerosis.

### 3.4 Neuropsychiatric disorders in relation to the degree of disability

Regarding to MS, neuropsychiatric disorders predominated in patients with mild disability, anxiety was presented in 41% (*n* = 48/117), follow by depression in 34% (*n* = 40/117). In NMOSD, psychiatric comorbidities were presented in patients with moderate disability, anxiety in 50% (n = 17/34), follow by depression in 61.7% (*n* = 21/34). However, no statistically significant differences were found in relation to the degree of disability in any of the categories (Table 4).

**Table 4:** Neuropsychiatric disorders in relation to the degree of disability.

Neuropsychiatric disorders	No or mild disability	Moderate disability	Severe disability	*p* value
	EDSS 0–3,5	EDSS 4–6	EDSS > 6,5	
	*N* (%)	*N* (%)	*N* (%)	
MS *n* = 117				
Anxiety severity				
Mild	25 (21.4%)	12 (10.3%)	2 (1.7%)	0.93
Moderate/severe	23 (19.7%)	11 (9.4%)	2 (1.7%)	0.96
Depression severity				
None	36 (30.8%)	13 (11.1%)	3 (2.6%)	0.67
Mild	23 (19.7%)	12 (10.3%)	2 (1.7%)	0.86
Moderate	15 (12.8%)	4 (3.4%)	2 (1.7%)	0.46
Severe	2 (1.7%)	5 (4.3%)	-	0.03
Chronic alcoholism	-	1 (0.9%)	-	0.15
Borderline personality disorder	-	-	1 (0.9%)	0.002
Adjustment disorder	4 (3.4%)	2 (1.7%)	-	0.81
Specific phobia	-	1 (0.9%)	-	0.29
Insomnia	4 (3.4%)	1 (0.9%)	-	0.73
Bipolar disorder	2 (1.7%)	-	-	0.58
Suicidal tendency	-	-	-	-
NMOSD *n* = 34				
Anxiety severity				
Mild	3 (8.8%)	3 (8.8%)	2 (5.9%)	0.19
Moderate/severe	2 (5.9%)	14 (41.2%)	4 (11.8%)	0.36
Depression severity				
None	1 (2.9%)	6 (17.6%)	2 (5.9%)	0.84
Mild	4 (11.8%)	8 (23.5%)	4 (11.8%)	0.41
Moderate	1 (2.9%)	3 (8.8%)	1 (2.9%)	0.99
Severe	-	4 (11.8%)	-	0.26
Chronic alcoholism	1 (2.9%)	1 (2.9%)	-	0.44
Borderline personality disorder	-	-	-	-
Adjustment disorder	-	1 (2.9%)	1 (2.9%)	0.54
Specific phobia	1 (2.9%)	-	-	0.09
Insomnia	1 (2.9%)	1 (2.9%)	1 (2.9%)	0.58
Bipolar disorder	1 (2.9%)	-	-	0.09
Suicidal tendency	-	2 (5.9%)	-	0.54

NMOSD, Neuromyelitis Optica Spectrum Disorders; MS, Multiple Sclerosis; EDSS, Expanded Disability Status Scale.

## 4 Discussion

This is the first study on psychiatric disorders in Ecuadorian patients with demyelinating disorders through the use a well-validated structured psychiatric interview. In general, we found that the most frequent neuropsychiatric pathology in both MS and NMOSD was anxiety followed by depression. The presence of neuropsychiatric disorders in demyelinating diseases such as MS and NMOSD can accelerate the progression of the disease, increase hospitalizations and reduce the ability to cope with its physical symptoms, therefore the treatment of these disorders involves a multidisciplinary approach that includes medical treatment, psychological and psychiatric support.

The prevalence of psychogenic or functional disorders may be common in Neurology, there may be a tendency to over diagnoses of these disorders in patients who suffer from demyelinating diseases, therefore it is important to correctly evaluate the patient during the first year that they have been diagnosed so that they receive appropriate care. Appropriate psychological and psychiatric treatment if required and can be treated in time (25).

The presence of anxiety in MS patients is variable, in our study we found a frequency of 70%. However, our previous report we found a lower prevalence (25%) (26). A meta-analysis also found a lower prevalence of anxiety in MS patients with 22% (9). A possible explanation of this variable frequency is based on the test that were used which is not the same between the studies. In our study, it is important to note that the frequency of moderate and severe anxiety was higher in NMOSD in comparison with MS (82% vs. 70% respectively). Ziyan Shi et al. (27) demonstrated that 68% of the NMOSD patients had anxiety. In contrast, a previous study from Unite Kingdom showed that anxiety was presented in 13% of the patients in MS and 8% in NMOSD and possible explanation of this difference is the size of the sample which was small (5).

In our study we found that depression was frequent in both MS and NMOSD with 31.6 and 47% respectively, these data are very similar to other reports around the word. Moore et al. (5) demonstrated that lifetime prevalence of depression was 46 and 34% for MS and NMOSD, respectively. Fernández et al. (6) showed in patients from Argentina that mild depression predominated in both diseases MS and NMOSD with 30 and 11%, respectively. Similarly, Ayzenberg et al. (10) showed in 166 NMOSD patients that depression was present in 39.8%; 19.3% of them presented mild depression, 11.6% moderate depression and 7.8% severe depression. Ziyan et al. (27) demonstrated in 73 NMOSD patients that depression was present in 25%. Finally, in the Brazilian study carried out by Mendes et al. The frequency of depressive and anxiety symptoms in patients with relapsing–remitting Multiple Sclerosis (RRMS) was 17.9 and 34.5% respectively; these studies found a positive correlation between the degree of depression and disability (28). The prevalence of depression in MS our study is very similar in comparison with Ecuadorian type II diabetes mellitus (DM) patients in which one study found a prevalence of depression of 31.7%. In contrast in NMOSD patients the prevalence of depression in our study was higher than type II DM patients (47% vs. 33.7%). It can be explained by the severity of disability that NMOSD produces (29).

The areas of the brain that are associated with organic depression are the prefrontal area, the hippocampus, the limbic system, which are preserved in NMOSD, in the case of MS there are lesions at the level of the gray matter and these areas are correlated with depression. Longer disease duration was associated with higher levels of agoraphobia, somatization in the case of NMOSD, while in MS patients it was associated with higher levels of depression, anxiety, panic attacks, phobia, obsessive-compulsive disorder, of post-traumatic stress, paranoia, suicide (30, 31). In our study, patients with a longer duration of illness were associated with anxiety and depression.

It should be noted that the prevalence of suicide is higher in patients with demyelinating diseases that in general population. Fernández et al. (6) showed that 30% of NMOSD patients had attempted suicide at least once during the course of the disease. In our study we found that the prevalence of attempted suicide in NMOSD was lower (5.9%) in comparison with the study of Fernandez. It is important to note that in our study we did not find suicide attempt in MS patients. A British study has showed that attempted suicide was more frequent in NMOSD than in MS patients (41% vs. 5% respectively) (5). A study from China which included 569 NMOSD patients showed that 8.3% of them died by suicide due to depression caused by severe attacks or sustained disability (32). There is not a clear explanation why the frequency of attempted suicide was low in our patients in comparison with data from other studies. In a previous study from Ecuador found that male, adolescents and low education was associated with attempted suicide these findings were not present in our cohort. In addition, in Ecuador suicide is frequent in rural areas and provinces of amazon region these contrast with our MS and NMOSD patients which come from urban areas and the majority living in Quito a city of Sierra region (33) Finally, although international studies report the prevalence of psychotic symptoms in MS and NMOSD at around 0.41 to 7.46%, in our study no cases were found in the two demyelinating diseases (4).

The prevalence of drug abuse in patients with demyelinating disorders is from 2.5 to 7.4%, the main drug abuse is related to the use of cannabis to control neurological symptoms. However, no cases were identified in our patients (6). In the case of alcohol abuse, the prevalence is higher in comparison with drug abuse between 3.9 to 18.2% in MS (6), in our patients we found alcohol abuse in 0.8 and 5.9% for MS and NMOSD respectively, indicating a low prevalence of consumption of these substances and very similar to reported in other studies.

In this study, statistically significant associations between neuropsychiatric disorders based on age and degree of disability were not found, although it should be noted that the largest number of cases was found in patients between the ages of 41 and 50, both in MS and in NMOSD. No significant association was found between the higher degree of disability and the increase in neuropsychiatric disorders such as anxiety or depression. Silvera et al. (4) demonstrated in 205 patients with MS a statistically significant association between depression, anxiety and EDSS disability and disease course in both RRMM and SPMS.

Bipolar disorder in patients with NMOSD is a complex issue, since both conditions can influence each other, either due to the direct effects of the disease on the central nervous system or derived from the use of chronic treatments. In predisposed patients, the use of drugs can trigger manic or hypomanic episodes. In our study, bipolar disorder occurred in 1.7% of patients with MS and 2.9% with NMOSD, borderline personality disorder in 0.9% in MS. Chavarro, et al. stated that the level of somatic symptoms was higher in patients with NMOSD; likewise, somatization was statistically significantly associated with depression in these patients (34, 35).

Finally, our study has a number of limitations. Our patients are come from a clinical population attending a specialist neurosciences hospital rather than from a community which places limitations on the generalizability of our data. NMOSD is a rare condition around the word it is the reason why the main challenge is to achieve large sample sizes that would increase confidence in the finding. Finally, our examination of neuropsychiatric symptoms was largely exploratory and involved comparison of a number of variables. Future research in MS and NMOSD patients from LATAM might usually examine specific aspects of mood disorders in this population.

To conclude in our study, we found a high prevalence of neuropsychiatric disorders in a cohort of Ecuadorian patients with demyelinating diseases especially anxiety and depression, the frequency was higher in NMOSD than MS. While the prevalence of suicide was low in our study and very similar to reported in other studies, more studies are necessary specifically in NMOSD an uncommon disease in which there are not enough mood disorders studies.

## Data Availability

The raw data supporting the conclusions of this article will be made available by the authors, without undue reservation.
